# Obesity and Insulin Resistance in Asthma Pathogenesis and Clinical Outcomes

**DOI:** 10.3390/biomedicines12010173

**Published:** 2024-01-12

**Authors:** Sabina Škrgat, Matevž Harlander, Miodrag Janić

**Affiliations:** 1 Department of Pulmonary Diseases and Allergy, University Medical Centre Ljubljana, SI-1000 Ljubljana, Slovenia; matevz.harlander@kclj.si; 2 Medical Faculty, University of Ljubljana, SI-1000 Ljubljana, Slovenia; miodrag.janic@kclj.si; 3 Clinical Department of Endocrinology, Diabetes and Metabolic Diseases, University Medical Centre Ljubljana, SI-1000 Ljubljana, Slovenia

**Keywords:** insulin resistance, obesity, asthma, inflammation, therapeutic options, challenges

## Abstract

Common inflammatory ground links obesity, insulin resistance, and asthma. As recognition of their interplay, one worsening the natural course of the other, is recognised, questions remain about how to adequately address them altogether to improve clinical outcomes. The present manuscript sheds light on the problem, describing possible pathophysiological links, clinical views, and therapeutic challenges, raising questions about what remains to be done, and calling for multidisciplinary treatment of these patients to detect diseases early and adequately address them before they become full-blown and deteriorate their health and quality of life.

## 1. Introduction

Obesity is reaching the prevalence of pandemics, in addition to associated comorbidities. It induces metabolic derangements that lead to the aggravation of present comorbidities and, therefore, affects pre-existing diseases. The prevalence of asthma is also increasing. Furthermore, it has been concluded that overweight (BMI ≥ 25 kg/m^2^) and obese (BMI ≥ 30 kg/m^2^) people have 1.5–2.5-fold increased risk of developing asthma compared to their lean counterparts [1]. Obesity leads to various metabolic disorders, including dysglycaemia, insulin resistance, and diabetes, among others. People with diabetes have a 2.2-fold increased risk of developing asthma [2]. The increase in risk is independent of allergy. The presence of obesity in asthma patients leads to poorer control of asthma with persistent respiratory symptoms, increased use of control and rescue medications, and systemic glucocorticoids, leading to a lower quality of life for these patients. In addition, obese asthma patients are more prone to severe asthma. They have an increased burden of emergency room visits and hospitalisations, including admissions to intensive care units [1,2,3]. Taken together, it can be concluded that obesity, with its metabolic environment also leading to insulin resistance, affects asthma in a detrimental way.

## 2. Obesity and Asthma Connections

The connections between obesity and asthma are not fully understood. However, several links have been hypothesised: common etiological and predisposing factors, mechanical changes in the respiratory system, comorbidities, chronic systemic inflammation, endocrine factors, and metabolic syndrome, including insulin resistance [3]. The general environment common to both states is low-grade inflammation, characterised by peripheral leukocytosis and elevated C-reactive protein (CRP) in serum. Adipokines are produced by adipose tissue and activate the innate immune response in adipocytes, perpetuating inflammation. The volume of adipose tissue determines the volume of inflammation induced. The inflammatory environment caused by adipokines has detrimental effects on the systemic cellular and functional organs, including the lungs [4]. These adipokines modulate airway inflammation and bronchial hyperresponsiveness. Pro-inflammatory adipokines are leptin, resistin, tumour necrosis factor α (TNF-α), monocyte chemoattractant protein-1 (MCP-1), and interleukin-6 (IL-6), while the anti-inflammatory adipokine is adiponectin. In obesity, there is a disturbed balance between pro-inflammatory and anti-inflammatory adipokines. There is an abundance of pro-inflammatory leptin that is supposed to reduce energy balance, but obese people usually develop resistance to leptin. It induces inflammation, particularly via interferon-mediated responses, as well as via increased CD4+ T cell immunity and activation of mast cells in addition to lung fibroblasts [5]. Smooth muscle cells of the airways express leptin receptors. The mechanism of direct leptin effects on airways is not fully understood. Leptin signalling appears to regulate the diameter of the airways independently of its effect on appetite and energy metabolism. On the one hand, there are data that it contributes to the narrowing of the airways. On the other hand, it is speculated that leptin might act on airway smooth muscle cells via neural mechanisms. It has been hypothesised that leptin-induced bronchodilation could occur via parasympathetic signalling via M3 muscarinic receptors in smooth muscle cells of the airways [6]. This means that its action might be controversial. On the other hand, the effect of adiponectin is reduced, and thus is its effect on reducing IL-6 and TNF-α. The releases of IL-10 and Il-1 are also reduced [7,8]. Adiponectin acts on the lungs via several specific receptors that are expressed in smooth muscle cells of the airways. Although it plays a role in the immune response, its direct active role in smooth muscle cells of the airways has not yet been elucidated [5]. Summarising these effects leads to the activation of helper T cells, particularly helper T cells 17, which are responsible for neutrophilic airway inflammation; they also induce chemotaxis and activation of leukocytes and monocytes. In addition to the effects described, there is also an increase in oxidative stress in obese asthma patients [1,4,5,7,8].

Obesity is associated with reduced lung volumes, mainly due to abdominal and thoracic adipose tissue that prevents normal lung inflation. A higher BMI was associated with a reduced forced expiratory volume in 1 s (FEV1) and a reduced forced vital capacity (FVC) according to some studies, while others did not find this connection or the effects were very small and, therefore, not worthy of recognition. However, it seems that the data are uniform in relation to the decrease in expiratory reserve volume (ERV), which has been found to be much larger and to have a clear dose–response relationship according to BMI. Due to tidal breathing with low tidal volumes and frequent collapses of small airways in this setting, this can increase the damage to already affected airways in asthma, thus strengthening the already present pro-inflammatory environment. However, obesity per se has been determined to add very little to the presence and severity of hyperresponsiveness of the airways in asthma [1]. 

Misdiagnosis of asthma is common, but not more so in obese patients than in non-obese patients. Aaron et al. conducted a prospective study of 540 individuals with physician-diagnosed asthma and, after evaluation with bronchial reversibility, methacholine challenge, and withdrawal of asthma medication, they concluded that 31.8% of obese patients and 28.7% of non-obese patients diagnosed with asthma were misclassified as having asthma [9,10]. 

## 3. Early Mechanisms and Links between Asthma and Obesity

Being overweight or obese is associated with an increased risk of new-onset asthma in children. These early derangements could begin already in utero, with evidence that children whose mothers were obese during pregnancy were at higher risk of asthma or wheezing [11]. In a later longitudinal study of 12,963 children and adolescents and their mothers, Dumas et al. found that high maternal pre-pregnancy BMI and low birth weight were associated with an elevated risk of asthma in the offspring. On the other hand, gestational diabetes was not associated with asthma in the offspring [12]. The mechanisms involved may include inflammatory changes during pregnancy: (a) metabolic alterations and dysregulation of the balance of reactive oxygen species in the mother-placenta-foetus unit and (b) influences on the programming of the neonatal immune system that could provide a possible link to an increased incidence of chronic inflammatory diseases such as asthma later in life [13,14].

The findings of Egan et al. in 1596 Norwegian adolescents and young adults aged 12–30 years showed that the presence of general or abdominal obesity is a risk factor for the development of asthma during 11 years of follow-up. They also showed a bidirectional temporal association between asthma and general and abdominal weight status, suggesting that asthma is a risk factor for subsequent overweight and obesity [15].

## 4. Molecular Regulation of Crucial Cells Involved in Obesity-Associated Asthma

One of the important challenges in the era of personalised medicine is understanding the molecular mechanisms behind various clinical phenotypes of the disease, including obesity-associated asthma. Therefore, the identification of the relevant molecular endotypes would enable researchers to establish highly detailed methods of molecular endotyping. This would allow the identification of asthmatics who benefit the most from specific treatment options [16,17]. CD4+ T cells are known to play an important regulatory role in asthma development by orchestrating the actions of a variety of effector cells [18,19]. Alhamdan et al. explored the specific role of CD4+ T cells in obesity-associated low type-2 asthma. They compared the transcriptome profiles of these cells in strictly selected, clinically characterised obese and non-obese low type-2 asthmatics. RNA sequencing (RNA-Seq) was carried out on peripheral blood CD4+ T cells and thousands of differentially expressed genes were identified in both asthma groups compared to heathy controls. The expression of the interferon (IFN) stimulated gene associated with IFN-related signalling pathways was specifically affected in obese asthmatics. Despite the small sample, these results might signal towards therapeutic strategies targeting the IFN signalling pathway or some of its central genes. This could represent a personalised specific treatment option for low-type-2 obese low type-2 asthmatics [20].

Epigenetics is a heritable characteristic that affects gene expression without altering the DNA sequence [21]. Environmental factors in the prenatal period, such as maternal smoking and other factors after birth, such as traffic or air pollution, nutrients, and drugs, could be trigger factors for epigenetic changes [22]. Epigenetic modification occurs during prenatal development, early childhood, and adolescence, as these are the periods in life when people are susceptible to various asthma triggers [23]. DNA methylation, post-translational histone modifications, and microRNA (miRNA) expression are the most commonly identified epigenetic mechanisms and play a regulatory role in immune responses and gene expression in asthma [24,25]. In a very recent study, Alhamdan et al. explored additional extracellular vesicle (EV) miRNA signatures to gain new insights into the underlying molecular mechanisms of obesity-associated low type-2 asthma. Integrative pathway analysis of potential target genes of differentially expressed miRNAs revealed that inflammatory cytokines and metabolic factors were specifically related to obesity-associated low type-2 asthma. In plasma EV miRNA signatures, they identified two miRNA groups, miR-17-92 and -106a–363, to be associated with specific inflammatory and metabolic mechanisms of the obesity-associated low type-2 asthma phenotype. Together with several canonical signalling pathways, including IL-6, TGF-β, and IFN signalling in conjunction with their associated EV miRNAs, these results support the concept that obesity asthma represents a unique asthma phenotype with quite specific underlying pathomechanisms. Therefore, their observations can be used as a solid basis for future research, especially in the development of targeted therapy concepts for this asthma phenotype [26].

## 5. Insulin Resistance and Asthma

### 5.1. Molecular Background

The dysmetabolic environment in obesity includes disease states such as dyslipidaemia, hyperglycaemia, and insulin resistance. Insulin resistance originates from the deposition of free fatty acids in the liver and the consequent induction of gluconeogenesis that leads to compensatory insulin hypersecretion from the pancreas. Additionally, free fatty acids decrease muscle sensitivity to insulin, which leads to reduced glucose uptake into muscles. Obesity and adipokines released from adipose tissue aggravate insulin resistance and lipolysis of triglycerides in adipose tissue and the release of free fatty acids, concluding a vicious cycle characterised by hyperinsulinemia levels and low-grade inflammation [27,28]. Hyperinsulinemia has an important impact on airway hyperresponsiveness. It affects nerve function. Hyperinsulinemia induces hyperresponsiveness of the parasympathetic nerves that are crucial in controlling airway bronchoconstriction. This is a compensatory effect in insulin resistance. It is due to the high insulin concentration that increases neuronal acetylcholine release via the disruption of the presynaptic inhibitory M2 muscarinic receptors on parasympathetic nerves. The loss of M2 function in addition to increased acetylcholine release increases bronchoconstriction [29]. On the other hand, insulin also induces transactivation of a G-protein coupled receptor kinase 2-dependent β2 adrenergic receptor cascade in airway smooth muscle cells that leads to reduced accumulation of cyclic adenosine monophosphate and, therefore, to impaired relaxation of airway smooth muscle cells [30]. Furthermore, hyperinsulinemia of insulin resistance is associated with α-smooth muscle actin and peribronchial collagen deposition, as well as increased levels of β-catenin. These suggest the proliferation of airway smooth muscle cells and the potentially irreversible pro-constrictive and profibrotic effects of insulin resistance in the lungs [31].

Insulin resistance is associated with glucose intolerance. Glucose induces oxidative stress that additionally increases inflammation. This hyperglycaemia-induced damage can last long after glucose levels are normalised, as there is the so-called ‘metabolic memory’, which has been observed in cells such as adipocytes [32]. 

In addition, lipids associated with obesity, which are in part responsible for insulin resistance, also induce inflammation in adipose tissue by activating toll-like receptors (TLRs). These can be activated via a variety of dietary factors in response to obesity-induced metabolic stress. TLRs sense free fatty acids, ceramides, other proteins, and modified low-density lipoproteins. These substances also activate TLRs, in particular TLR2 and TLR4. The signal is transduced via the MyD88-dependent and MyD88-independent pathways that activate the nuclear factor κ-light-chain-enhancer of activated B cell (NF-κB) and MAPK pathways. These then inhibit insulin signalling via serine phosphorylation of the insulin receptor substrate (IRS). Furthermore, these activated pathways induce the transcription of pro-inflammatory cytokines: TNF-α, IL-6, pro-IL-1, and pro-IL-18. Activators of TLRs also activate Nod-like receptor-protein 3 (NLRP3), probably via the induction of reactive oxygen species. NLRP3 then assembles with the adaptor protein, an apoptosis-associated speck-like protein containing a caspase-recruitment domain (ASC) and caspase-1 in a multiprotein complex called the inflammasome, which cleaves the inactive precursors of pro-IL-1 and pro-IL-18 to the active forms of IL-1 and IL-18. In addition, long-chain free fatty acids, which are chronically elevated in obesity, directly activate the inflammasome in airway smooth muscle cells, where they activate the free fatty acid receptor 1 (FFAR1). This leads to activation of phospholipase C and extracellular signal regulated kinase signalling cascade, resulting in intracellular calcium deposition and consequent bronchoconstriction, as well as airway smooth muscle cell proliferation, both aggravating asthma symptoms [33,34,35,36].

### 5.2. Functional Background

Cross-sectional analysis, using a community-based prospective cohort in Korea, showed that FVC was significantly lower in the insulin resistance subset, and prospective analyses showed that both FEV1 and FVC declined faster in those with insulin resistance [37]. Furthermore, in an analysis using data from the Severe Asthma Research Programme 3 (SARP-3), Peters and colleagues evaluated the relationships between the homeostatic measure of insulin resistance (HOMA-IR) and lung function [38]. They found that most of the cohort was obese and that almost half had insulin resistance. The overlap in the subgroups with obesity and insulin resistance was not complete, with approximately a quarter of the insulin resistance subgroup not having obesity and approximately a third of the obese participants not having insulin resistance. One of the main highlights of the study was that cross-sectional analyses of FEV1 and FVC values from 3-year visits were lower in participants with insulin resistance than in those without insulin resistance. Insulin resistance was independently associated with low lung function in asthma; the effects of body mass on the mechanics of the chest wall were unlikely to explain this association. The decrease in FEV1 in patients with insulin resistance was approximately 25–30 mL per year higher than in those without insulin resistance, showing an accelerated decline in lung function beyond the decline expected with normal ageing [38,39]. There was also a significantly worse FEV1 response to albuterol beta agonist in patients with severe insulin resistance and a significantly worse systemic corticosteroid response test (SCRT) response to intramuscular triamcinolone acetonide (TA) in patients with moderate and severe insulin resistance. Importantly, participants with insulin resistance had a low percentage of sputum eosinophils, a marker of type 2 airway inflammation [38]. As can be seen, insulin resistance only reaffirms the connection between obesity and asthma [40].

Additionally, an emerging research area appears to be the investigation of lung function trajectories over time. The longitudinal characterisation of obstruction and restriction patterns, as well as their overlap from early life to later adulthood, showed that individuals with a restrictive pattern only (low FVC only) had evidence of true lung restriction later in life and were at risk for multiple morbidities, including obesity and diabetes [41]. It seems that an early approach to improving lung function, diminishing the restrictive longitudinal lung function pattern, could be a target strategy to combat the high prevalence of comorbidities in later life. 

## 6. Molecular Links between Insulin and Airway Inflammation in Obesity-Associated Asthma

**Figure 1:**
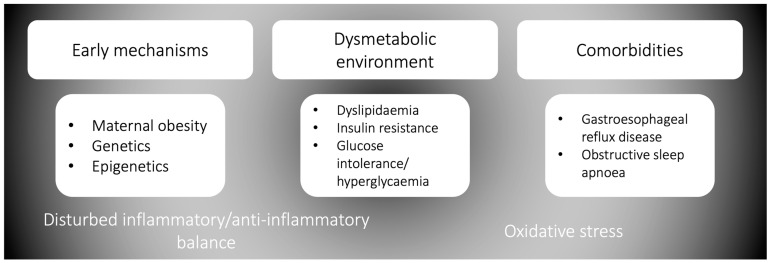
The elements and complexity of the obesity-associated asthma phenotype.

Hyperinsulinemia in the lung has not yet been broadly evaluated; thus, knowledge of its consequences in the lung is lacking. Hyperinsulinemia per se may have adverse effects on the structure and function of the airways according to data derived from murine models. Insulin-induced contractile effects on smooth muscle cells of the airways may be causally related to the development of an asthma-like phenotype [31]. In another murine model, the authors observed that obesity induced by a high-fat diet increased insulin resistance and the expression of transforming growth factor-β1 (TGF-β1) in the lungs, leading to peribronchial and perivascular lung fibrosis and increased airway hyperresponsiveness. Importantly, they highlighted that the bronchial epithelium could produce TGF-β1 via insulin stimulation [42]. TGF-β1 might be the connection between airway remodelling and airway hyperresponsiveness in obesity-associated asthma, as shown in Figure 1 [43]. TGF-β is an adipokine with different roles in maintaining cellular homeostasis, lung development, and physiology [44]. TGF-β stimulates fibroblast connective tissue production, and increased expression of TGF-β in the lungs can induce tissue dysfunctions, such as the airway remodelling seen in asthma and chronic bronchitis [43,44,45]. TGF-β can be secreted by epithelial cells, fibroblasts, eosinophils, and mast cells [45]. Importantly, inflammation and tissue remodelling with pathological fibrosis are common consequences of Th2 responses in the lung and other organs. Interleukin-13 (IL-13) and TGF- β1 are frequently coexpressed in these responses, which play an important role in the pathogenesis of Th2-induced pathologies. Studies have shown that IL-13 mediates its fibrogenic effects, at least in part, via its ability to induce and activate TGF- β1 [46,47]. Finally, some studies have reported that TGF-β1 is associated with insulin resistance [48], which is a critical problem in obesity and is associated with pro-inflammatory reactions involving various immune cells and cytokines [49]. The mechanisms of obesity-associated inflammatory lung diseases, such as asthma, are only partially understood. Taking into account the evidence that TGF-β1 acts as a key modulator, future novel therapies could target TGF-β1 signalling pathways to improve clinical asthma outcomes [50]. However, the role of TGF-β1 may be complicated, as some investigators have also reported that TGF-β1 has anti-inflammatory effects on allergic asthma [51]. Hyperinsulinemia can also aggravate bronchoconstriction via vagal stimulation and reduction in the inhibitory function of the M2 muscarinic receptor without changes in the contractility of airway smooth muscle cells to acetylcholine [52].

## 7. Clinical View of Asthma and Obesity

Asthma and obesity are undoubtedly related, but the true relationship among them is not clearly understood. It could be that obesity affects the development and severity of asthma and could also affect its control. However, its association as a driving force for development, a confounder, or a comorbidity in asthma remains to be established. It is a complex process depicted in Figure 1. The subgrouping of asthma patient phenotypes is based on clinical characteristics, triggers, or general inflammatory processes, or response to therapy. Consequently, the phenotypes of obese asthma patients have also been identified [53,54]. 

Several obesity-associated asthma phenotypes have been identified. First, it has been found particularly in women with late-onset asthma and with a “type-2 low” inflammation that it turns out to be associated with resistance to conventional asthma treatment and inhaled glucocorticoid treatment, as explained below. Subjects appear to have little or no eosinophilic inflammation; therefore, its main drivers appear to be changes in the structure and function of the airways [21,22,23,55]. An obesity-associated asthma phenotype discovered in the National Institutes of Health Severe Asthma Research Programme (SARP) by Moore et al. was found to be characteristic primarily for older women (mean age 50 years; range 34 to 68 years) with the highest BMI (58% with BMI > 30) and late-onset asthma (all older than 23 years). This subpopulation was less likely to be atopic [56] and turned out to have a decreased baseline lung function (71% with FEV1 < 80% of predicted), although its duration of asthma was shorter. These subjects reported a significant oral glucocorticoid load, as 17% of the patients received regular systemic corticosteroids. They also reported an increase in the use of healthcare care (particularly the need for oral corticosteroid bursts) and daily asthma symptoms. The clinical characteristics of this group highlighted the relationship between obesity, the level of asthma symptoms, and the use of health care [54].

Another (second) subgroup (phenotype) of obesity-associated asthma begins with early eosinophilic inflammation and elevated serum IgE levels [21,22,23,53,54,55]. In the SARP cohort described above, this subgroup had worse asthma control than non-obese asthmatics, characterised by a three-fold increase in hospitalisations and a six-fold increase in intensive care admissions compared to non-obese asthmatics. These subjects were also more likely to have continuous symptoms (wheezing, nocturnal symptoms), consequently a lower quality of life, and generally more severe asthma compared to early-onset leaner subjects with asthma [54,57].

Scott et al. proposed a third subgroup, the neutrophilic obesity-associated asthma phenotype. These subjects have an increase in neutrophilic airway inflammation and a distinct difference depending on sex. It turns out that late-onset neutrophilic asthma is more characteristic for obese women, who had airway neutrophils >61% compared to non-obese asthmatic women (42.9% vs. 16.2% *p* = 0.017). Additional characteristics include elevated serum levels of IL-6 and IL-17 sputum levels in association with an increase in neutrophil count. Interleukin-17 has been associated with poor asthma control and worsening lung function. More studies are needed to better define this phenotype and establish its clinical characteristics [23,54,58].

## 8. Asthma, Obesity, and Their Comorbidities

### 8.1. Obstructive Sleep Apnoea (OSA)

The prevalence of obstructive sleep apnoea (OSA) and asthma is increasing. However, OSA and asthma have adverse effects on each other with different interactive mechanisms under upper and lower airway pathologies, in addition to shared comorbidities such as obesity, rhinitis, and gastro-oesophageal reflux [59,60]. The prevalence of OSA among asthmatic populations has been reported to be 38 to 70% [60]. One of the main reasons is structural and collapsable changes in the upper airways during sleep related to the use of inhaled corticosteroids (ICS), oral corticosteroids, and systemic corticosteroids [60,61].

The features of OSA, particularly chronic intermittent hypoxia (CIH), may lead to inflammation and/or remodelling of the lower airways that may increase asthma morbidity. CIH increases airflow obstruction [62]. OSA causes chronic systemic inflammation with activation and release of cytokines and inflammatory mediators such as TNF-a, IL-6, vascular endothelial growth factor (VEGF), pentane, 8-isoprostane, CRP, leptin, and matrix metallopeptidase-9. Many studies have found that OSA can lead to increased neutrophils in the sputum, which are associated with increased type 1 airway inflammation, airway remodelling, steroid resistance, and increased severity of the disease in asthma [63,64,65].

Previous studies have reported that OSA is one of the most important pathophysiological mechanisms related to the worsening of asthma symptoms and control, in addition to shared risk factors and comorbidities [66]. However, in a recent meta-analysis, Wang and colleagues aimed to examine the relationship between obstructive sleep apnoea and asthma severity. OSA was associated with more severe or more difficult-to-control asthma with decreased %FEV1 and increased airway inflammation, especially in children. %FEV1 tended to decrease in adult patients with OSA but did not reach statistical significance. Asthma increased daytime sleepiness but did not significantly worsen the severity of OSA despite the lack of a subgroup for the severity of asthma. More studies are needed to investigate the effect of asthma on the severity of OSA and the impact of different OSA on the prevalence of asthma. Overall, clinicians should perform early detection, diagnosis, and therapy of OSA in patients with asthma to slow the rate of airway remodelling and the decrease in lung function [59].

### 8.2. Gastro-oesophageal Reflux Disease (GERD)

Asthma and GERD are common clinical conditions, but their frequent presence as comorbid conditions raise the possibility of shared biological mechanisms underlying these two diseases [54]. Epidemiological data suggest that GERD occurs in 30–80% of asthmatic subjects [67]. Oesophageal manometry and 24 h oesophageal pH tests demonstrate frequent GERD in the absence of obvious symptoms in stable asthmatics [68]. Denlinger and colleagues found an increased rate of asthma exacerbations among those with GERD [69].

There are two proposed mechanisms by which GERD impacts asthma severity and exacerbations: micro-aspiration of gastric contents causing airway inflammation, respiratory symptoms, and lung injury, and vagal nerve stimulation that occurs from reflux of acidic gastric contents into the lower oesophagus leading to bronchoconstriction and respiratory symptoms. Furthermore, bronchoconstriction and hyperinflation in asthma can induce acid reflux due to changes in the pressure gradient between the abdomen and the chest that change the tone of the lower oesophageal sphincter (LES) [70,71,72,73]. 

The results of the clinical trials of acid suppression on asthma outcomes are inconsistent. Littner et al. found that lansoprazole twice daily improved overall quality of life and reduced asthma exacerbations in subjects with symptomatic reflux, with the most pronounced effect occurring in more severe asthmatics [70,74].

The interplay between asthma, obesity, GERD, and OSA is not fully understood. Although not studied collectively, the identification and treatment of these comorbidities has a positive impact on asthma control [70].

## 9. Interventions

As obesity, insulin resistance, and asthma interconnect, the question is how the influence of each of these entities affects the other. There is the aspect of asthma treatment, on the one hand, and weight loss, as well as reduction of insulin resistance, on the other. First, the treatment of asthma in obese patients is more difficult and does not respond adequately to inhaled glucocorticoid therapy as in lean individuals [1]. This may be due to different types of airway inflammation related to obesity-associated asthma. Anderson et al. performed a post hoc analysis of a cross-over study investigating the efficacy of inhaled budesonide in 72 patients with asthma divided into two groups: lean (BMI < 25 kg/m^2^) and overweight (BMI ≥ 25 kg/m^2^). Patients were treated for 4 weeks with 200 mg/day or 800 mg/day budesonide. Although no differences were found with respect to the change in FEV1 and bronchial hyperresponsiveness between lean and overweight asthma patients, inhaled budesonide was less effective in improving symptoms in overweight asthma patients [1,75]. There is also evidence of a high oral corticosteroid burden in obese asthmatics. The findings of Gibeon et al., who compared the clinical characteristics of obese (BMI ≥ 30 kg/m^2^) and non-obese (BMI 18–25 kg/m^2^) patients with severe asthma, have shown that despite a similar degree of airflow obstruction and eosinophilic sputum inflammation, obese asthma patients received maintenance treatment more often with a high dose of oral prednisolone [76]. This approach is unlikely to improve the non-responsive corticosteroid factors related to reduced asthma control, but it may lead to side effects that can be quite severe, among others the fact that it drives insulin resistance and, consequently, obesity [1]. An approach that should be avoided at all costs. On the other hand, there is evidence that montelukast is as effective as it is in lean individuals [1,3,77].

From 70 to 80% of patients with corticosteroid naive asthma and 50% with corticosteroid-treated asthma are estimated to have an elevated sputum eosinophil count [78], which is generally associated with enhanced expression of the type-2 cytokines interleukin (IL) -4, IL-5, and IL-13 [79], increased fractional exhaled nitric oxide (FeNO), and peripheral blood eosinophilia. This “type-2 high” phenotype is characteristically responsive to inhaled corticosteroid treatment and, in severe disease, to biologic agents targeting these type-2 cytokines. These include anti-IgE omalizumab, anti-IL-5 antibodies (mepolizumab, reslizumab), anti-IL-5 receptor antibody (benralizumab), and anti-IL-4 receptor antibody (dupilumab) [80]. There remains a significant proportion of people with “type-2 low”, a phenotype frequently linked to obesity. The personalised approach in severe forms of asthma includes different approaches. Several studies suggested that the long-term use of macrolide antibiotics may have antisteroid-sparing or anti-inflammatory effects in asthma [81]. The AMAZES study that randomly assigned 420 adults with moderate to severe asthma to 500 mg or placebo three times a week for 48 weeks showed a striking reduction in asthma exacerbations, and, unexpectedly, a subgroup analysis found azithromycin effective in both eosinophilic and non-eosinophilic phenotypes [82]. Thymic stromal lymphopoietin (TSLP) is an epithelial-cell-derived cytokine implicated in the initiation and persistence of inflammatory pathways in asthma. Treatment with anti-TSLP (tezepelumab) decreased the frequency of asthma exacerbations regardless of the asthma phenotype, including patients with eosinophil-low asthma. It improved lung function in patients with asthma and decreased airway hyperresponsiveness to mannitol in clinical trials [83].

The other side of the coin represents weight loss, as well as the treatment of insulin resistance and diabetes and their effects on asthma. Few studies have investigated the effects of weight loss on measures of airway inflammation [1]. According to a study by Dixon et al., bariatric surgery did not improve airway inflammation, as shown by the number of inflammatory cells in the bronchoalveolar lavage fluid [84]. Huisstede et al. investigated the effects of profound weight loss after bariatric surgery on lung function and systemic inflammation, as well as inflammatory cell counts in bronchial biopsies in 27 asthmatics and 39 non-asthmatics with morbid obesity. Improvements in symptoms and large and small airway function were observed in obese participants with and without asthma. Furthermore, in patients with asthma, there was a marked improvement in bronchial hyperresponsiveness [85]. Furthermore, better asthma control, better quality of life, and improved lung function were also observed [3]. The response to treatment in obese asthmatics is altered, as well as a consequence of obesity-associated comorbidities. Some, such as obstructive sleep apnoea and gastro-oesophageal reflux, can simulate asthma symptoms and, if misinterpreted, lead to an unnecessary increase in anti-asthmatic medications that do not result in clinical improvement [23,86].

Furthermore, pharmacological reduction in insulin resistance has been shown to be effective, as metformin, the most prescribed antidiabetic drug, reduced the burden of the disease by reducing IL-6-induced inflammation, oxidative stress, and insulin resistance. However, thiazolidinediones, drugs with proven effects in reducing insulin resistance, according to recent randomised trials, did not affect asthma burden [77]. Recently, new modern antidiabetic drugs are emerging, particularly glucagon-like peptide-1 receptor agonists, which have been shown to reduce insulin resistance, control glycaemia, and lead to significant weight loss regardless of the presence of diabetes. Their effect on asthma has been shown in obese people, as their receptors are present in the airways. They appear to reduce airway inflammation and mucus secretion directly in the lungs. However, the efficacy in obese asthma is also expected due to its significant effect on weight reduction, as weight loss of 5% to 10% has been shown to be associated with better asthma control [1,3,27,77]. Furthermore, sodium-glucose cotransporter-2 inhibitors (SGLT-2) have been shown to induce relaxation of pulmonary smooth muscle cells, also paving the way for their repurposing in these settings of metabolic and airway diseases [87]. However, it should be prudent to interpret these interventional studies using new antidiabetic drugs, as there are probably multiple factors, in addition to the antidiabetic drugs used, that lead to clinical benefits (study environment, optimal anti-asthmatic therapy, etc.).

**Figure 2:**
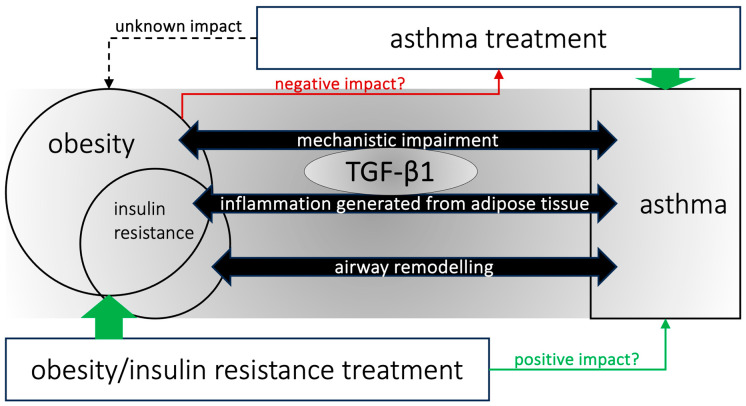
Interplay between obesity, insulin resistance, and asthma with therapeutic implications, also showing the centralised role of transforming growth factor-β1 (TGF-β1).

The interrelationship between obesity, insulin resistance, and asthma with therapeutic implications is represented in Figure 2.

## 10. Challenges

As the field of common ground of comorbidities, asthma, obesity/insulin resistance, and diabetes is emerging, there is a new polygon for more intensive research that would lead to improved clinical outcomes. There is a lack of robust research that addresses the common molecular pathways between these disease states that would allow adequate therapeutic address. In terms of diagnostics, better and more precise diagnostic methods that would recognise the disease of small respiratory pathways implicated in these states would be required, as spirometry is not sensitive enough to detect early changes. And in obese patients/insulin-resistant patients, screening for the involvement of small respiratory pathways would benefit earlier interventions and probably less deterioration of lung disease. Furthermore, the effects of interventions would need to be evaluated in detail, as emerging therapies in the fields of obesity/diabetes allow their repurposing in many states of inflammatory diseases. The concept emerges that with early treatment with recent, very effective metabolic therapies (i.e., SGLT-2 inhibitors, GLP-1 receptor agonists, and probably also new dual or triple co-agonists), the burden of asthma could be reduced by preventing its appearance, progression, or aggravation, particularly in those with “type-2 low” phenotypes, while increasing the efficacy of drugs and reducing potential side effects in those with “type-2 high” phenotypes. And, not least, the multidisciplinary treatment of patients with asthma and obesity/insulin resistance/diabetes is of the most importance, as ensuring the treatment of all aspects of the disease, respiratory and metabolic, would lead to better clinical results and translate into a better quality of life for our patients.
